# Behavior and Regional Cortical BOLD Signal Fluctuations Are Altered in Adult Rabbits After Neonatal Volatile Anesthetic Exposure

**DOI:** 10.3389/fnins.2020.571486

**Published:** 2020-10-23

**Authors:** Alexander Drobyshevsky, Mike J. Miller, Limin Li, Conor J. Dixon, Palamadai N. Venkatasubramanian, Alice M. Wyrwicz, Daniil P. Aksenov

**Affiliations:** ^1^Department of Pediatrics, NorthShore University HealthSystem, Evanston, IL, United States; ^2^Department of Radiology, NorthShore University HealthSystem, Evanston, IL, United States

**Keywords:** resting state fMRI, connectivity, thalamocortical, isoflurane, classical conditioning

## Abstract

Neonatal and infant exposure to volatile anesthetics has been associated with long-term learning, memory, and behavioral deficits. Although early anesthesia exposure has been linked to a number of underlying structural abnormalities, functional changes associated with these impairments remain poorly understood. To investigate the relationship between functional alteration in neuronal circuits and learning deficiency, resting state functional MRI (rsfMRI) connectivity was examined in adolescent rabbits exposed to general anesthesia as neonates (1 MAC isoflurane for 2 h on postnatal days P8, P11, and P14) and unanesthetized controls before and after training with a trace eyeblink classical conditioning (ECC) paradigm. Long-range connectivity was measured between several key regions of interest (ROIs), including primary and secondary somatosensory cortices, thalamus, hippocampus, and cingulate. In addition, metrics of regional BOLD fluctuation amplitudes and coherence, amplitude of low-frequency fluctuation (ALFF), fractional ALFF (fALFF), and regional homogeneity (ReHo) were calculated. Our results showed that the trace ECC learning rate was significantly lower in the anesthesia-exposed group. No anesthesia-related changes in long-range connectivity, fALFF, or ReHo were found between any ROIs. However, ALFF was significantly higher in anesthesia-exposed rabbits in the primary and secondary somatosensory cortices, and ALFF in those areas was a significant predictor of the learning performance for trace ECC. The absence of anesthesia-related changes in long-range thalamocortical connectivity indicates that functional thalamocortical input is not affected. Higher ALFF in the somatosensory cortex may indicate the developmental disruption of cortical neuronal circuits after neonatal anesthesia exposure, including excessive neuronal synchronization that may underlie the observed cognitive deficits.

## Introduction

Increasing concern exists regarding the potential of anesthesia exposure during infancy and early childhood to produce pathogenic effects on the developing brain that persist into adolescence and adulthood (Taylor, 2009; Sun, 2010; Crosby and Davis, 2013; Olsen and Brambrink, 2013; Lee et al., 2015). As approximately 6 million children in the United States undergo anesthesia (DeFrances et al., 2007) each year during surgeries, imaging studies, and other diagnostic procedures (Sun, 2010), including more than a million receiving multiple anesthesia exposures, this issue has a great clinical significance. While a number of structural changes have been identified that may link anesthesia and learning disability (Jevtovic-Todorovic et al., 2003), including cell loss in both the cortex and the hippocampus in infant animals (Jevtovic-Todorovic et al., 2003; Fredriksson et al., 2007; Istaphanous et al., 2011), functional changes in the brain associated with the learning and memory impairments remain poorly understood. Only a limited number of functional MRI (fMRI) studies were used to evaluate functional brain changes in response to neonatal anesthesia exposure (Aksenov et al., 2016, 2020b; Chen et al., 2019).

Neonatal anesthesia exposure could impact the cortical BOLD response either by causing altered input to the cerebral cortex or by inducing abnormal maturation of cortical circuits, including changes in synaptic plasticity and disturbances of the excitatory/inhibitory balance. In the latter case, such disturbances would affect the intracortical neurovascular unit, which regulates local blood flow through direct and indirect neurovascular interactions (Muoio et al., 2014). Functional alterations in long-range thalamocortical or local intracortical networks can be evaluated using long- and short-range functional connectivity resting state MRI (rsfMRI) approaches (Monti et al., 2013). Indeed, the mapping of slow BOLD signal oscillations was used in multiple studies involving brain development (Vasung et al., 2019; Zhang et al., 2019). rsfMRI measures, such as amplitude of low-frequency fluctuation (ALFF) and regional homogeneity (ReHo), are considered to be highly reproducible (Golestani et al., 2017). Low-frequency fluctuations have been suggested to reflect the intensity of regional spontaneous brain activity (Zou et al., 2008). While most of the developmental rsfMRI studies focus on the strengthening and expansion of long-range functional connectivity during brain maturation, a characteristic developmental trajectory has been described also for short-range connectivity (Ouyang et al., 2017). rsfMRI can be easily used in clinical practice due to minimal requirements for subject cooperation, and thus this method has significant translational value, especially for pediatric patients. As widespread neuronal injury has been reported in the hippocampus, cortex, and thalamus in animal models of neonatal inhaled anesthetic exposure (Patel and Sun, 2009), we hypothesized that rsfMRI would reveal alterations in long-range functional connectivity between the thalamus and the cortex due to the effects of neonatal anesthesia on thalamocortical input, as well as changes in short-range functional connectivity due to disrupted intracortical processing. We utilized a non-sedated rabbit model of neonatal isoflurane exposure (Aksenov et al., 2016, 2020b) that allowed us to avoid the confounding influence of anesthesia during imaging.

Our results showed that long-range thalamocortical functional connectivity in awake adult rabbits was not affected by neonatal anesthesia exposure. However, the local ALFF in the somatosensory cortex significantly increased in the anesthesia group, and this effect was significantly associated with changes in learning rate in anesthesia-exposed subjects.

## Materials and Methods

### Animal Model of Neonatal Anesthesia-Induced Brain Injury

This study was performed in accordance with the National Institutes of Health guidelines, and the NorthShore University HealthSystem Research Institute Institutional Animal Care and Use Committee approved the protocol. The study consisted of 12 rabbits and two groups: neonatal anesthesia exposure and controls, with 6 animals per group. The model and animal procedures have been described in detail previously in Reference (Aksenov et al., 2016). Briefly, newborn Dutch-Belted rabbits (three females and three males from three litters) underwent 1 MAC isoflurane (Abbot, IL, United States) anesthesia in air via nose cone mask on postnatal days 8, 11, and 14 for 2 h each exposure. The anesthetic dose was confirmed using a tail-clamping technique. Animal temperature was maintained at 35°C using a water blanket. The control rabbit kits (three females and three males from three litters) were exposed to the same environment as the anesthesia group while breathing room air. The rabbit kits were nursed with the dam until 4–6 weeks, which is the optimal weaning age for Dutch-Belted rabbits.

At the age of 3 months, animals were implanted with a restraining head bolt and habituated to the MRI environment as described previously (Aksenov et al., 2016). Animals were anesthetized with a mixture of ketamine (60 mg/kg) and xylazine (10 mg/kg). Then, 5–6 small bur holes were made in the skull without full penetration of the bone. Small nylon support screws were inserted into the bur holes. A lightweight head-restraining device containing four nylon bolts was implanted on top of the skull. This device also served to position the radiofrequency (RF) coil in the stereotaxic plane during MRI experiments. After 1 week of recovery after surgery, each rabbit was habituated for 3–5 days to the imaging environment prior to the experiments in the MRI scanner.

### Functional MRI With Whisker Stimulation and Resting State Functional MRI Data Acquisition

All MRI experiments were conducted on awake rabbits. MRI experiments were performed on a 9.4 T imaging spectrometer (BioSpec 94/30USR; Bruker BioSpin MRI GmbH, Germany) with an actively shielded gradient coil (BFG-240-150-S-7; Research Resonance, Inc., Billerica, MA, United States). A single-turn, 20-mm-diameter circular RF surface coil was used for both transmission and reception. Prior to each experiment, anatomical images were acquired using a multislice gradient echo pulse sequence with a repetition time (TR) of 1.5 s, an echo time (TE) of 10 ms, a 30 mm × 30 mm field of view (FOV), and a matrix size of 128 × 128, corresponding to an in-plane resolution of 234 μm × 234 μm. Functional MRI (fMRI) data were acquired from four consecutive axial slices using a single-shot, gradient echo multislice echo-planar imaging (EPI) sequence with a TR of 2 s, an TE of 11 ms, a 30 mm × 30 mm FOV, and a matrix size of 80 × 80, reconstructed to an in-plane pixel size of 234 μm × 234 μm using zero filling, and a 1-mm slice thickness with no gap. Whisker stimulation was delivered by deflecting two whiskers (A1 and B1) at an amplitude of 1.5 mm and a frequency of 50 Hz using a system described previously (Li et al., 2012; Aksenov et al., 2016). Each animal received 10 trials of whisker stimulation, which consisted of baseline (30 s), stimulation (20 s), and poststimulus (40 s) periods, before and after training with the eyeblink conditioning paradigm, as described below. After the stimulation paradigm, resting state scan was obtained with the same imaging parameters and slice geometry as the stimulation scan, except the number of slices was extended to 8, and 450 volumes were acquired during 15 min.

### Functional MRI Data Processing and Analysis

**FIGURE 1:**
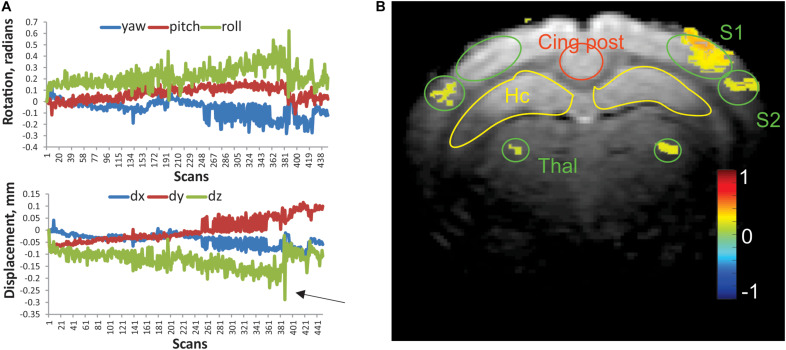
**(A)** Representative linear displacement and rotational motion in a resting state scan of a control rabbit, determined from image realignment procedure. Arrow indicates a frame with excessive relative motion, excluded from analysis. **(B)** Activation map of the whisker stimulation paradigm in an individual rabbit. Activated voxels were determined by cross-correlation of BOLD signal with stimulation time course. The color bar represents Pearson correlation coefficient. Regions of interests (ROIs) of predefined shape were placed bilaterally on the primary and secondary somatosensory cortices (S1 and S2), ventral posterior thalamus (Thal), hippocampus (Hc), posterior cingulate (Cing post), and anterior cingulate (2 mm rostral from this slice).

To ensure accurate delineation of anatomical regions involved in the whisker barrel sensory pathway, processing of fMRI and placement of region of interests (ROIs) were performed in individual animal space. The fMRI data were stripped of the skull and corrected for small head motion using a 3-D affine registration method implemented in AIR 5.0 (Woods et al., 1998), and the subsequent processing was implemented in MATLAB (The MathWorks, Inc., Natick, MA, United States). Individual frames with relative displacement of more than half the voxel size in-plane were excluded from analysis, and the missing frames were interpolated with cubic spline. Ten initial volumes were discarded to account for signal stabilization. For the whisker stimulation paradigm, the time course of the BOLD signal for each voxel was de-trended, smoothed in-plane with Gaussian full width at half maximum (FWHM) = 1 mm, and cross-correlated with a box-car whisker stimulation time course. A conservative threshold of 0.15 for Pearson correlation was used to identify activated voxels during the stimulation paradigm (Figure 1). Circular ROIs of predetermined fixed size were placed at the centers of activated clusters in the contralateral (right) primary (S1) and secondary (S2) somatosensory cortexes and dorsolateral thalamus (Thal) for each individual animal. If not detected by the stimulation paradigm, homologous ROIs were placed on the opposite side in the mirror location relative to the midline. In addition, ROIs were drawn over the hippocampi bilaterally, posterior cingulate (Cing post) at the same plane as S1, and anterior cingulate (Cing ant) 2 mm rostral. ROIs were transferred to the resting state scans.

For rsfMRI, realigned and de-trended scans were temporally filtered with a 0.1-Hz low-pass filter and spatially smoothed as described above. Global image intensity and signal in ventricles were regressed out as nuisance covariates. Pearson correlation coefficients between delineated ROIs were obtained as a measure of long-range functional connectivity and Fisher’s z-transformed for group comparisons. As measures of regional BOLD fluctuation and coherence within studied ROIs, we used indexes of ALFF, fractional ALFF (fALFF), and ReHo, calculated with a MATLAB routine from the REST toolkit (Song et al., 2011) (version plus V1.24)^1^. For this analysis, the preprocessing was as above, except no temporal filters were applied for ALFF and fALFF. After transforming voxel time series frequency information into the power domain, ALFF is calculated as the sum of amplitudes within a low-frequency range (0.01–0.08 Hz), which is proportional to the original ALFF implementation (Zang et al., 2007), derived from power spectrum. fALFF is intended to reduce the sensitivity of ALFF to physiological noise by taking the ratio of power spectrum in low-frequency band (0.01–0.08 Hz) to the total frequency range (0–0.25 Hz). For ReHo calculation, time series were band-pass filtered (0.01–0.08 Hz), and no spatial smoothing was applied. ReHo (Zang et al., 2004) is defined as the connectivity of a given voxel to those of its nearest 18 neighboring voxels using Kendall’s coefficient of concordance.

### Eyeblink Classical Conditioning

Eyeblink classical conditioning (ECC) is a well-controlled test commonly used in both animals and humans, in which the subject learns to associate a neutral conditioned stimulus (CS) with a behaviorally salient unconditioned stimulus (US). The CS was delivered by deflecting two whiskers (A1 and B1) attached to a fiber band on the rabbit’s left side at an amplitude of 1.5 mm and a frequency of 50 Hz, using a system described previously (Li et al., 2012). The US consisted of a 3-psi air puff to the left eye supplied by compressed air and controlled by a regulator and solenoid valve. Eyelid movements were measured with a fiber optic-based infrared reflectance sensor. The durations of the CS and US were 250 and 150 ms, respectively, with 500 ms stimulus-free trace interval. The total duration of a single trial was 8 s, with a 5- to 10-s random intertrial interval. A conditioned response (CR) was defined as a change in the voltage from the detector that was 4 SD greater than the mean baseline amplitude and occurred at least 35 ms after the onset of the CS but before the US. Each subject received one session of conditioning trials per day for 10 days, where each session consisted of 100 trials. Percent of CRs at day 10 is reported as a measure of learning ability.

### Statistical Analysis

Fisher’s z-transformed Pearson correlation coefficients of the time courses of BOLD signal between ROIs and metrics of regional BOLD fluctuation amplitudes and coherence, averaged within each ROI, were used to determine mean group differences with Student’s *t*-test. *P*-values of ROI-to-ROI tests were corrected for multiple comparisons using a false discovery rate (FDR) procedure (Benjamini and Hochberg, 1995), implemented in MATLAB. Difference in percent of CR between the control group and the neonatal anesthesia exposure group was tested with two-sample unpaired Student’s *t*-test. Relationships between performance in eyeblink conditioning test and fMRI indexes were tested with linear regression model using Microsoft Excel. Differences were considered significant at *p*-values ≤ 0.05.

Power analysis for the difference of means^2^ indicated that with group sizes *n* = 6 for each group, type I error rate α = 0.05, type II error rate β = 0.2, and standard deviation for the control group of 0.17 for the thalamus–primary sensory cortex ROI-to-ROI normalized correlation coefficient, detected effect size would be 22.7% of the mean value. Similarly, for ALFF in S1, standard deviation for the control group of 0.12, detected effect size would be 26.7% of the mean value; for fALFF in S1, standard deviation for the control group of 0.08, detected effect size would be 24.3% of the mean value; for ReHo in S1, standard deviation for the control group of 0.15, detected effect size would be 37.2% of the mean value.

## Results

### Whisker Stimulation Identifies Thalamocortical Circuit in Individual Animals in Non-sedated Rabbit

Habituated rabbits tolerated MRI imaging procedures (Miller et al., 2003) with minimal head motion due to the implanted head bolt. Typical motion parameters of a functional scan are presented in Figure 1A. Absolute displacement rarely exceeded the in-plane voxel size (0.2 mm). The numbers of scans that exceeded the predetermined threshold for absolute displacement (arrow in Figure 1A) per functional run were 3.4 ± 1.5 and 4.2 ± 1.8 for the control and anesthesia groups, respectively. These values were not significantly different (*t*-test, *p* = 0.875). The whisker stimulation paradigm used in this study reliably identified the activated areas in the contralateral primary (S1) and secondary somatosensory (S2) barrel cortices and ventral posterior nuclei of the thalamus as shown in Figure 1B. While activated voxels were detected in the ipsilateral thalamus and S2 for this animal, activated voxels were rarely found in ipsilateral S1 with this paradigm, and ROIs were placed in a mirror position of the contralateral side.

### Thalamocortical Connectivity Is Not Different After Neonatal Anesthesia as Compared With Controls

**FIGURE 2:**
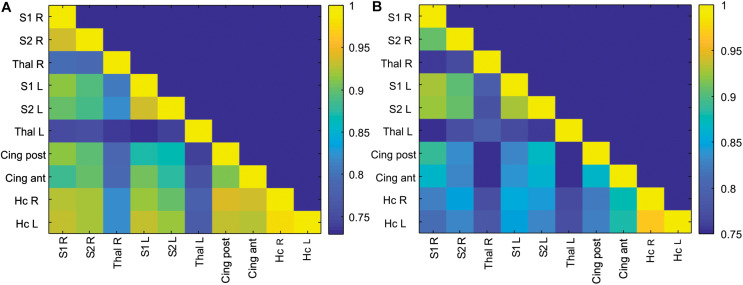
Correlation matrix of ROI-to-ROI connectivity in the control **(A)** and anesthesia **(B)** groups. Color bars represent values of Pearson correlation coefficients.

Correlation matrices of z-transformed Pearson correlation coefficients between studied ROIs were calculated for the control and anesthesia groups (heat maps in Figure 2). While overall decrease in long-range connectivity is apparent in the anesthesia group (as indicated by more blue squares in Figure 2A), no significant differences between the somatosensory cortices and the thalami were found after multiple comparison corrections, contrary to our primary hypothesis.

### Alteration in Regional BOLD Signal Fluctuations and Coherence Measures After Neonatal Anesthesia Exposure

**FIGURE 3:**
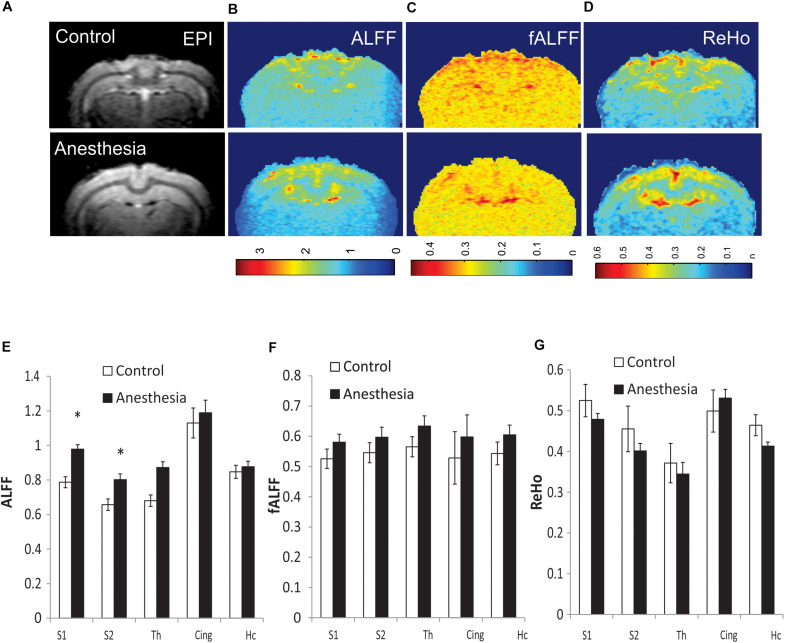
Representative maps of regional resting state functional (rsfMRI) BOLD fluctuations and coherence in individual animals: amplitude of low-frequency fluctuation (ALFF) **(B)**, fractional ALFF (fALFF) **(C)**, and regional homogeneity (ReHo) **(D)**, shown with corresponding functional echo-planar imaging (EPI) image **(A)**. Top row—control rabbit, bottom row—rabbit after neonatal anesthesia exposure. Mean values of ALFF **(E)**, fALFF **(F)**, and ReHo **(G)** for selected ROIs, averaged between the left and right sides, *p < 0.05.

Due to the global nature of injury induced by neonatal anesthesia, and in order to reduce the number of comparisons, values of short-range connectivity indices were averaged between the left and right sides. Representative maps of calculated ALFF, fALFF, and ReHo for individual rabbits in the control and anesthesia groups are shown in Figures 3A–D. ALFF was significantly increased in the anesthesia group in S1 (*p* = 0.009, *p*_*FDR*_ = 0.026) and S2 (*p* = 0.011, *p*_*FDR*_ = 0.026), as shown in Figure 3E. fALFF and ReHo did not change significantly (Figures 3F,G). Paired *t*-test did not reveal any differences between the left and right sides of analyzed structures including the somatosensory cortex.

### Association of Learning Deficits in Eyeblink Classical Conditioning With Regional Resting State Functional MRI Measures

**FIGURE 4:**
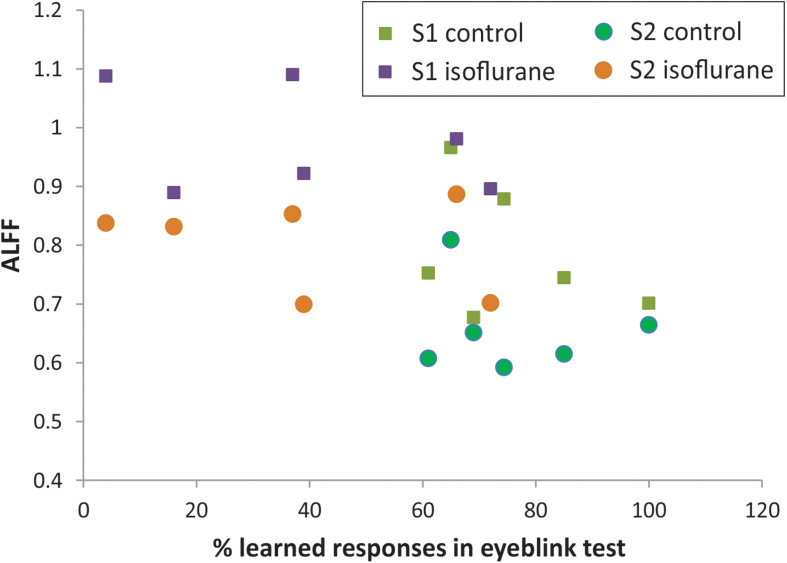
Relationship between ALFF in S1 and S2 and memory function on classical eyeblink conditioning test.

The percent of CRs was significantly lower in the group with neonatal anesthesia exposure than in controls: 39.0 ± 10.9 vs. 75.7 ± 5.9, *t*-test, *p* = 0.014, as previously reported (Aksenov et al., 2016). To explore the predictive value of regional BOLD fluctuation indices for learning performance, linear regression models were tested with S1 and S2 in ALFF as predictors. Although the anesthesia and control groups were effectively separated using rsfMRI, ALFF, and behavioral eyeblink learning variables (Figure 4), no significant relationship between the variables was found within the groups either in S1 or in S2 regions.

## Discussion

Comparing adult animals exposed to anesthesia as neonates with unanesthetized controls allowed us to assess the long-term effects of anesthesia on rsfMRI with the focus on thalamocortical connectivity. Two major findings emerged from this study: the absence of alterations in long-range thalamocortical connectivity and elevated amplitudes of low-frequency BOLD fluctuations in S1 and S2 in rabbits with neonatal exposure to isoflurane relative to controls.

Detecting disturbances in thalamocortical connectivity during development is of great interest since such disruptions may underlie sensory, motor, and cognitive abnormalities that manifest later (Alcauter et al., 2014; Ball et al., 2015). While the development and alterations of structural and functional thalamocortical connectivity have been examined in human infants (Zhang et al., 2010; Smyser et al., 2011), there is a paucity of such studies in animal models of brain injury during development. Utilizing sensory whisker stimulation, we identified functional thalamocortical connections and used functionally connected areas as ROIs. Surprisingly, no significant differences between the control and anesthesia-exposed groups in long-range ROI-to-ROI connectivity between the somatosensory cortices and the thalamic nuclei were found (Figure 2). Several possible explanations can account for the absence of changes in thalamocortical connectivity. For example, the functional interaction between the thalamus and the cortex was maintained without significant change after neonatal anesthesia exposure due to insufficient injury or compensatory changes, or that changes between the experimental groups were subtle enough as to be undetectable by rsfMRI. The former possibility is supported by the lack of changes in BOLD signal shape in the anesthesia-exposed group in our previous study (Aksenov et al., 2016).

Widespread damage of the cerebral cortex and thalamus following neonatal anesthesia exposure was reported previously. For example, after a 3-h exposure to isoflurane, apoptosis was observed in the somatosensory, visual, and auditory cortices (Noguchi et al., 2017). Another study (Kodama et al., 2011) described neuronal death in layer IV of the sensory cortex after exposure to 4 or 8% desflurane for 6 h in P6 mice. Similar damage, although to a lesser degree, was also observed after 2% isoflurane and 3% sevoflurane. Mice exposed to isoflurane at P3 exhibited an acute neuroapoptotic response in the thalamus (Maloney et al., 2019). Exposure to propofol at P6 in rhesus macaques (Creeley et al., 2013) also resulted in damage to the thalamus, but this damage was less severe compared with the cerebral cortex.

Despite these previous reports of cellular injury in the cortex and thalamus, we found no difference in the magnitude and shape of the BOLD signal in the somatosensory cortex with whisker stimulation in either controls or anesthesia-exposed rabbits (Aksenov et al., 2016), which would indicate that the pathological effect of injury was not pronounced, at least on the functional level. A recent review (Aksenov et al., 2020a) concluded that the functions of the sensory systems are affected less by neonatal anesthesia than learning-related functions due to the stronger ability of the sensory system to compensate for significant structural damage. For example, the loss of 50% or more sensory fibers/neurons may not produce sensory deficiency. Thus, the intact thalamocortical connectivity that we observed indicates that neonatal anesthesia, at least under the conditions of our protocol, was not able to produce sufficient neuronal damage to overcome the functional compensatory abilities of the thalamocortical system. Alternatively, it is possible that the timing of the injury with respect to the development of the thalamocortical circuitry could play a role. The thalamus–sensorimotor and thalamus–salience connectivity networks are among the earliest to develop and are already present in human neonates and, therefore, may be less affected by anesthesia exposure, whereas the thalamus–medial visual and thalamus–default mode network connectivity networks emerge later at 1 year old (Alcauter et al., 2014) and, thus, may be more susceptible to anesthesia exposure.

The ability to detect changes in thalamocortical functional connectivity may also reflect the characteristics of the system hardware. For these experiments, we used a surface coil attached to the head bolt to image non-anesthetized rabbits, which is characterized by decreasing coil sensitivity in the inferior part of the brain. However, here as well as in our previous studies (Aksenov et al., 2019), we have been able to detect robust activation in both cortical regions and thalamic nuclei using somatosensory and visual stimulation. Rodent fMRI studies of somatosensory stimulation have commonly reported BOLD changes only in S1 cortex, which may be due to the effects of anesthetics and/or the low fMRI sensitivity in the thalamus (Jung et al., 2019). Our previous observations with sensory stimulation in awake, unanesthetized rabbits found that the magnitude of the BOLD response in the thalamus was actually larger than that in the cortex (Aksenov et al., 2019), confirming our ability to robustly detect BOLD response in the thalamus.

Physiological considerations may also play a role in the rsfMRI results. The thalamocortical network is not typically identified using independent component analysis in rsfMRI data in humans and animals (Becerra et al., 2011), including rabbits (Schroeder et al., 2016). At the same time, anatomically plausible parcelation of the cortex using seed-based correlation of the rsfMRI time course of atlas-delineated thalamic nuclei has been demonstrated in humans (Zhang et al., 2010) and in group analysis of adult rats (Liang et al., 2013). These results are consistent with known thalamocortical connectivity from tracing and electrophysiological studies. It should be noted that, in the absence of external stimulation, thalamocortical functional connectivity in rsfMRI in our data was much less than the connectivity between homologous bilateral regions of the cortex and hippocampus, as shown in Figure 2. Using a surface coil with less sensitivity in deep brain areas in our study may also affect amplitudes and noise levels in BOLD signal in the thalamus, although the ALFF was not dramatically lower in this area (Figure 3E). Therefore, while detection of alterations in thalamocortical functional connectivity is possible, it may be more difficult due to relatively lower magnitude of the BOLD signal changes. Additionally, as a limitation of the study, it should be noted that a relatively small number of animals was used in the experimental group because rabbits are expensive subjects requiring high maintenance and labor costs, although the power analysis showed that this number of animals is sufficient for this type of experiment, as indicated in the section “Materials and Methods.”

Notably, we found that ALFF in the somatosensory cortex significantly increased in the anesthesia group. ALFF has been used previously to characterize a variety of brain pathologies, including Alzheimer’s disease (Yang et al., 2018), stroke (Wang et al., 2020a), and Parkinson’s disease (Wang et al., 2020b). Since ALFF measures the total power for a given time course within a low-frequency range (Zhan et al., 2016), it can characterize the overall state of the regional vasomotion but cannot reliably distinguish between neuronal (Liao et al., 2019) or non-neuronal vasomotion (Auer, 2008) components. Neonatal anesthesia causes widespread damage of cortical neurons (Kodama et al., 2011; Creeley et al., 2013; Noguchi et al., 2017), which may result in abnormal local circuit formation and lead to over-synchronized neuronal activity (Hunt et al., 2013). This may manifest as increased ALFF, if fMRI low-frequency fluctuations are sensitive to neuronal synchronization (Chen et al., 2017).

A reduction of ALFF in the bilateral postcentral gyri has been reported in schizophrenia patients (Wang et al., 2019), whereas an increase in ALFF was found in the medial prefrontal cortex in adolescents with autism (Guo et al., 2017), which may reflect differences in connectivity and synchronization of activity in local cortical networks in these conditions. An increase in cortical ALFF in the neonatal anesthesia group may also indicate loss of inhibitory influence on neuronal activity and vasomotion provided by interneurons, which has been reported to be affected by neonatal anesthesia exposure (Zhou et al., 2011). On the other hand, ALFF also can be related to non-neuronal vasomotion. The dominant frequency of brain tissue oxygen oscillations (BTOO), which are attributed to vasomotion at the level of arterioles (Hudetz et al., 1998; Aalkjaer et al., 2011; Mateo et al., 2017), has been shown to change during development (Doubovikov and Aksenov, 2020). The dominant frequency of BTOO is around 1–2 cycles/min (0.0167–0.0333 Hz) in neonates (Aksenov et al., 2018) and approximately 10 cycles/min (0.167 Hz) in adults (Linsenmeier et al., 2016). Based on our findings, it is possible that neonatal anesthesia may disrupt normal developmental shift to higher frequency range of vasomotion, which could manifest as an increase in ALFF in adulthood.

The integrity of the somatosensory cortex is required for normal learning of trace eyeblink conditioning (Galvez et al., 2006), and damage to this area can affect conditioned responses. The observation of both increased ALFF in the somatosensory cortex and learning deficiency indicates that learning deficiency may depend not only on neuronal apoptosis but also on neurovascular damage (Kikuchi et al., 2017).

In summary, we identified changes in the functional MRI parameter ALFF that persisted into adolescence following neonatal anesthesia exposure. These findings may reflect both the initial effects of anesthesia exposure and subsequent development and compensatory changes within the neurovascular unit. Our results indicate that ALFF can be used as a biomarker of the damage and behavioral deficits induced by neonatal anesthesia.

## Data Availability Statement

The raw data supporting the conclusions of this article will be made available by the authors, without undue reservation.

## Ethics Statement

The animal study was reviewed and approved by the NorthShore University Health System’s Animal Care and Use Committee.

## Author Contributions

AD and DA wrote the manuscript. DA, LL, MM, and CD performed the experiments and analyzed the data. DA, AD, AW, and PV planned the experiments and interpreted the results. All authors approved the content and assisted in the revision and review of the manuscript.

## Conflict of Interest

The authors declare that the research was conducted in the absence of any commercial or financial relationships that could be construed as a potential conflict of interest.

## Footnotes

1 http://restfmri.net

2 http://sample-size.net
